# Bioactive Components in Fermented Foods and Food By-Products

**DOI:** 10.3390/foods9020153

**Published:** 2020-02-05

**Authors:** Vito Verardo, Ana Maria Gómez-Caravaca, Giulia Tabanelli

**Affiliations:** 1 Department of Nutrition and Food Science, University of Granada, Campus of Cartuja, 18071 Granada, Spain; 2 Institute of Nutrition and Food Technology ‘José Mataix’, Biomedical Research Center, University of Granada, Avda del Conocimiento sn., 18100 Armilla, Granada, Spain; 3 Department of Analytical Chemistry, University of Granada. Campus of Fuentenueva, Avda Fuentenueva s/n, 18071 Granada, Spain; 4 Department of Agricultural and Food Sciences, University of Bologna, Viale Fanin 42, 40127 Bologna, Italy

**Keywords:** food fermentation, food by-products, bioactive compounds, lactic acid bacteria, lycopene, phenolic compounds, biogenic amines, γ-aminobutyric acid (GABA)

Food fermentation is one of the most ancient processes of food production that has historically been used to extend food shelf life and to enhance its organoleptic properties. However, some research has demonstrated that it can also increase the nutritional value and/or digestibility of food.

Firstly, microorganisms, and in particular Lactic Acid Bacteria (LAB), besides their role in acidification, are able to produce huge amounts of secondary metabolites with excellent health benefits and preservative properties (i.e., antimicrobial activity). Indeed, some microorganisms can increase the levels of several bioactive compounds (e.g., vitamins, antioxidant compounds, peptides, etc.). Secondly, fermented foods contain living organisms that contribute to the modulation of the host’s physiological balance and gut microbiota, enriching, at the same time, the host’s diet with new bioactive molecules. 

Moreover, recent advances in fermentation are focused on food by-products; in fact, they are a source of potentially bioactive compounds that, after fermentation, could be used as ingredients for nutraceuticals and functional food formulations [1].

Because of this, understanding of benefits of food fermentation is a growing field of research in nutrition and food science.

This Special Issue aimed to present current knowledge and research trends concerning the use of fermentation technologies like the sustainable GRAS (Generally Recognized As Safe) process for food and nutraceutical production, to improve food quality. 

In this context, Verni et al. [2] reviewed the effect of fermentation on the antioxidant compounds of cereals and legume-derived foods. They reported that the ability of fermentation to improve food antioxidant properties is strictly related to the metabolic activities of the starter used. Briefly, the fermentation processes are able to improve the bio-accessibility of phenolic compounds. Moreover, bioactive peptides resulted from bacterial and fungal proteolysis.

Several original papers focused on the use of food by-product fermentation in order to improve the extraction of bioactive compounds. Doan et al. [3] isolated a filamentous fungus (*Clerodendron cyrtophyllum*) from the root of *Clerodendron cyrtophyllum* Turcz; they grew this fungus on isoflavones-rich soybean extract. Thanks to the high β-glucosidase production of *Clerodendron cyrtophyllum*, they were able to produce an isoflavones aglycones-rich soybean extract (e.g., genistein and daidzein), proposing this method for applications in the pharmaceutical and functional food industries. Another study carried out by Lordan and co-workers [4] assessed the antithrombotic activities of lipid extracts from brewing raw materials, by-products, wort, and beer. Briefly, they showed that the fermentation of a brewing industry’s by-products could play a key role in increasing the anti-platelet-activating factor bioactivity of polar lipids. Simat and co-workers [5] also proposed aquaculture by-products as an alternative source of sustainable and profitable bioactive fish oils. 

Tofalo et al. [6] studied the effect of traditional cheese fermentation on the accumulation of healthy (γ-aminobutyric acid or GABA) and toxic (biogenic amines (BA)) compounds. They confirmed a greater BA formation and proteolytic activity in cheese made by pig rennet than those made by calf and kid rennet. So, they proposed the selection of autochthonous amine-negative and amine-oxidizing LAB as a valuable strategy to decrease BA formation. However, high amounts of GABA were produced, and they were correlated with the use of ewe’s milk, time of ripening, and type of coagulant. 

Venturi and co-workers [7] selected two strains belonging to *Lactobacillus farciminis* and *Lactobacillus brevis* species and used them for amaranth bread production. Their results underlined the bread produced with these LAB showed higher antioxidant activity and total phenolic content compared to the control. Moreover, these strains were able to increase GABA concentration (up to 350%) in breads enriched with 20% amaranth flour.

Sevgili et al. [8] checked the use of different substrates to produce lycopene via *Blakeslea trispora* fermentation. They confirmed that the medium with natural oil showed more lycopene than the medium that contained only a carbon source. In fact, oils improved lycopene production and the highest lycopene concentration was obtained when the medium was added to sunflower and corn oils.

Finally, Fracassetti et al. [9] proposed a new analytical method for the simultaneous quantification of tryptophan (TRP), tryptophan ethylester (TEE), and melatonin isomers (MISs) in fermented foods, such as wine. A preconcentration of wine by Solid Phase Extraction (SPE) followed by high performance liquid chromatography (HPLC) analysis either with fluorescence or mass spectrometer detectors were applied. They suggested that this protocol could be a useful tool for monitoring the release of MEL and TEE, even when their concentration is very low (<0.02 µg/L), as well as to determine their presence and concentration during wine production and storage.

To conclude, the papers included in this Special Issue summarized the valuable use of fermentation technology to improve the content of bioactive compounds in foods and food by-products or to limit the presence of some toxic compounds, such as biogenic amines. At the same time, different analytical approaches were proposed in order to study the effect of fermentation on the food matrix and the possible metabolites that are produced. However, as suggested by several authors, more in vivo studies should be carried out to demonstrate the potential of fermentation to produce healthy foods.
